# Sustainable production of FAEE biodiesel using the oleaginous yeast *Yarrowia lipolytica*

**DOI:** 10.1002/mbo3.1051

**Published:** 2020-04-27

**Authors:** Aiqun Yu, Yu Zhao, Jian Li, Shenglong Li, Yaru Pang, Yakun Zhao, Cuiying Zhang, Dongguang Xiao

**Affiliations:** ^1^ State Key Laboratory of Food Nutrition and Safety, Key Laboratory of Industrial Fermentation Microbiology of the Ministry of Education Tianjin Key Laboratory of Industrial Microbiology Tianjin Engineering Research Center of Microbial Metabolism and Fermentation Process Control College of Biotechnology Tianjin University of Science and Technology Tianjin China

**Keywords:** biodiesel, biosynthesis, coculture, FAEE, metabolic engineering, Y. lipolytica

## Abstract

Fatty acid ethyl esters (FAEEs) are fatty acid‐derived molecules and serve as an important form of biodiesel. The oleaginous yeast *Yarrowia lipolytica* is considered an ideal host platform for the production of fatty acid‐derived products due to its excellent lipid accumulation capacity. In this proof‐of‐principle study, several metabolic engineering strategies were applied for the overproduction of FAEE biodiesel in *Y. lipolytica*. Here, chromosome‐based co‐overexpression of two heterologous genes, namely, *PDC1* (encoding pyruvate decarboxylase) and *ADH1* (encoding alcohol dehydrogenase) from *Saccharomyces cerevisiae*, and the endogenous *GAPDH* (encoding glyceraldehyde‐3‐phosphate dehydrogenase) gene of *Y. lipolytica* resulted in successful biosynthesis of ethanol at 70.8 mg/L in *Y. lipolytica*. The engineered *Y. lipolytica* strain expressing the ethanol synthetic pathway together with a heterologous wax ester synthase (MhWS) exhibited the highest FAEE titer of 360.8 mg/L, which is 3.8‐fold higher than that of the control strain when 2% exogenous ethanol was added to the culture medium of *Y. lipolytica*. Furthermore, a synthetic microbial consortium comprising an engineered *Y. lipolytica* strain that heterologously expressed MhWS and a *S. cerevisiae* strain that could provide ethanol as a substrate for the production of the final product in the final engineered *Y. lipolytica* strain was created in this study. Finally, this synthetic consortium produced FAEE biodiesel at a titer of 4.8 mg/L under the optimum coculture conditions.

## Graphical abstract

Several metabolic engineering strategies were successfully applied in this study towards direct fatty acid ethyl esters (FAEE) overproduction in the nonconventional oleaginous yeast *Yarrowia lipolytica*. Additionally, we examined the performance of a microbial consortium for its potential application in FAEE production. The results obtained in this work lay the foundation for future development of more efficient yeast cell factories for the sustainable production of FAEE biodiesel.

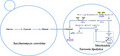

**Abbreviations**
FAEE: fatty acid ethyl esters
GAPDH: glyceraldehyde‐3‐phosphate dehydrogenase
GC/MS: gas chromatography–mass spectrometry
LB: Luria–Bertani
OD_600_: optical density at 600 nm
PCR: polymerase chain reaction
PDC: pyruvate decarboxylase
TCA cycle: tricarboxylic acid cycle
YPD: yeast extract–peptone–dextrose

## 1 INTRODUCTION

Currently, the demand for renewable and sustainable energy is rapidly increasing. Biodiesel is an important sustainable energy source, and fatty acid ethyl esters (FAEEs), popularly known as “biodiesel,” can serve as important alternative industrial diesel fuels. In the current global biodiesel market, most commercially available FAEEs are produced via a transesterification reaction between ethanol and various lipid feedstocks, such as plant oils or animal fats, in the presence of a catalyst (Dunn, Ngo, & Haas, 2015; Santana, Tortola, Reis, Silva, & Taranto, 2016; Suppalakpanya, Ratanawilai, & Tongurai, 2010). However, the major problems associated with this chemical synthesis method include the restricted availability of lipid sources and the risk of environmental pollution. In recent years, the use of genetically engineered microorganisms has provided a sustainable and environmentally friendly bioroute for the production of value‐added products, including biofuels, biochemicals, and other bioactive compounds (Cheon, Kim, Gustavsson, & Lee, 2016; Lee, Chou, Ham, Lee, & Keasling, 2008; Mao, Liu, Sun, & Lee, 2017; Marienhagen & Bott, 2013; Yu, Pratomo Juwono, Leong, & Chang, 2014).

The well‐known oleaginous yeast *Yarrowia lipolytica*, which can accumulate lipids at high amounts (Back, Rossignol, Krier, Nicaud, & Dhulster, 2016; Katre, Ajmera, Zinjarde, & Ravikumar, 2017), efficiently utilize a variety of low‐cost hydrophobic substrates for growth (Beopoulos, Chardot, & Nicaud, 2009; Fickers et al., 2010; Lopes, Gomes, Silva, & Belo, 2018), and produce various industrial enzymes and chemicals (Bankar, Kumar, & Zinjarde, 2009; Darvishi, Ariana, Marella, & Borodina, 2018; Ryu, Hipp, & Trinh, 2015; Yan et al., 2018; Zhu & Jackson, 2015), is fast becoming a promising microbial platform for various industrial applications. Moreover, recently, various molecular genetic tools and techniques have been designed and optimized to facilitate the genetic manipulation of *Y. lipolytica *(Holkenbrink et al., 2018; Schwartz, Hussain, Blenner, & Wheeldon, 2016; Wang, Hung, & Tsai, 2011; Yu et al., 2016) making this oleaginous yeast an ideal host for the engineering of high‐level production of fatty acid‐derived biofuels and biochemicals.

*Escherichia coli* was the first genetically engineered microorganism used for FAEE production, which was achieved by coexpression of the pyruvate decarboxylase gene and alcohol dehydrogenase gene from *Zymomonas mobilis* and the *atfA* gene encoding WS/DGAT (wax ester synthase/acyl‐coenzyme A: diacylglycerol acyltransferase) from *Acinetobacter baylyi* ADP1 (Kalscheuer, Stölting, & Steinbüchel, 2006). Further study revealed high wax ester synthase activity in *E. coli* with an enzyme from *Marinobacter aquaeolei* VT8 (MaWS1) from five tested WS/DGAT enzymes from four different bacteria, namely, *M. aquaeolei* VT8, *A. baylyi* ATCC 33305, *Rhodococcus jostii* RHA1, and *Psychrobacter cryohalolentis* K5. Thus, the WS from *M. aquaeolei* VT8 (MaWS1) showed the greatest potential for the heterologous production of wax esters and FAEEs in microbes (Barney, Wahlen, Garner, Wei, & Seefeldt, 2012). Moreover, the production of FAEEs by engineered *E. coli* cell factories was further enhanced upon adopting several metabolic engineering strategies and synthetic biology tools (Steen et al., 2010; Wierzbicki, Niraula, Yarrabothula, Layton, & Trinh, 2016).

To date, most studies have focused on metabolic engineering of the yeast *Saccharomyces cerevisiae* for FAEE production because the intracellular synthesis of FAEEs in *S. cerevisiae* can be easily achieved by heterologous expression of WS/DGAT using endogenously produced ethanol and fatty acyl‐CoA (or free fatty acids) as substrates (de Jong, Siewers, & Nielsen, 2016; Nielsen & Shi, 2020; Shi, Valle‐Rodríguez, Khoomrung, Siewers, & Nielsen, 2012; Shi, Valle‐Rodríguez, Siewers, & Nielsen, 2014; Thompson & Trinh, 2015; Valle‐Rodríguez, Shi, Siewers, & Nielsen, 2014; Yu, Jung, Kim, Park, & Han, 2012). It has also been proven that several wax ester synthases from different organisms, including *A. baylyi* ADP1, *Marinobacter hydrocarbonoclasticus* DSM 8798, *Rhodococcus opacus* PD630, *Mus musculus* C57BL/6, and *Psychrobacter arcticus* 273‐4, exhibit specific ester synthase activities, leading to the formation of FAEEs in *S. cerevisiae*. Of these enzymes, a wax ester synthase from *M. hydrocarbonoclasticus* DSM 8798 was found to be the best one for FAEE production (Shi et al., 2012).

However, in *S. cerevisiae*, the reported FAEE yield by heterologous expression of a wax ester synthase remains rather low because the production of FAEEs is greatly limited by the small pool of fatty acyl‐CoA and/or free fatty acids (Valle‐Rodríguez et al., 2014; Yu et al., 2012). Compared to the previously used *E. coli* and *S. cerevisiae* systems, the oleaginous yeast *Y. lipolytica* has outstanding lipid accumulation capacity. Thus, abundant intracellular fatty acyl‐CoA (or free fatty acids) is available for the production of FAEEs and other fatty acid‐derived bioproducts through this host's metabolic system (Abghari & Chen, 2014, 2017; Mlíčková et al., 2004; Tai & Stephanopoulos, 2013).

**FIGURE 1:**
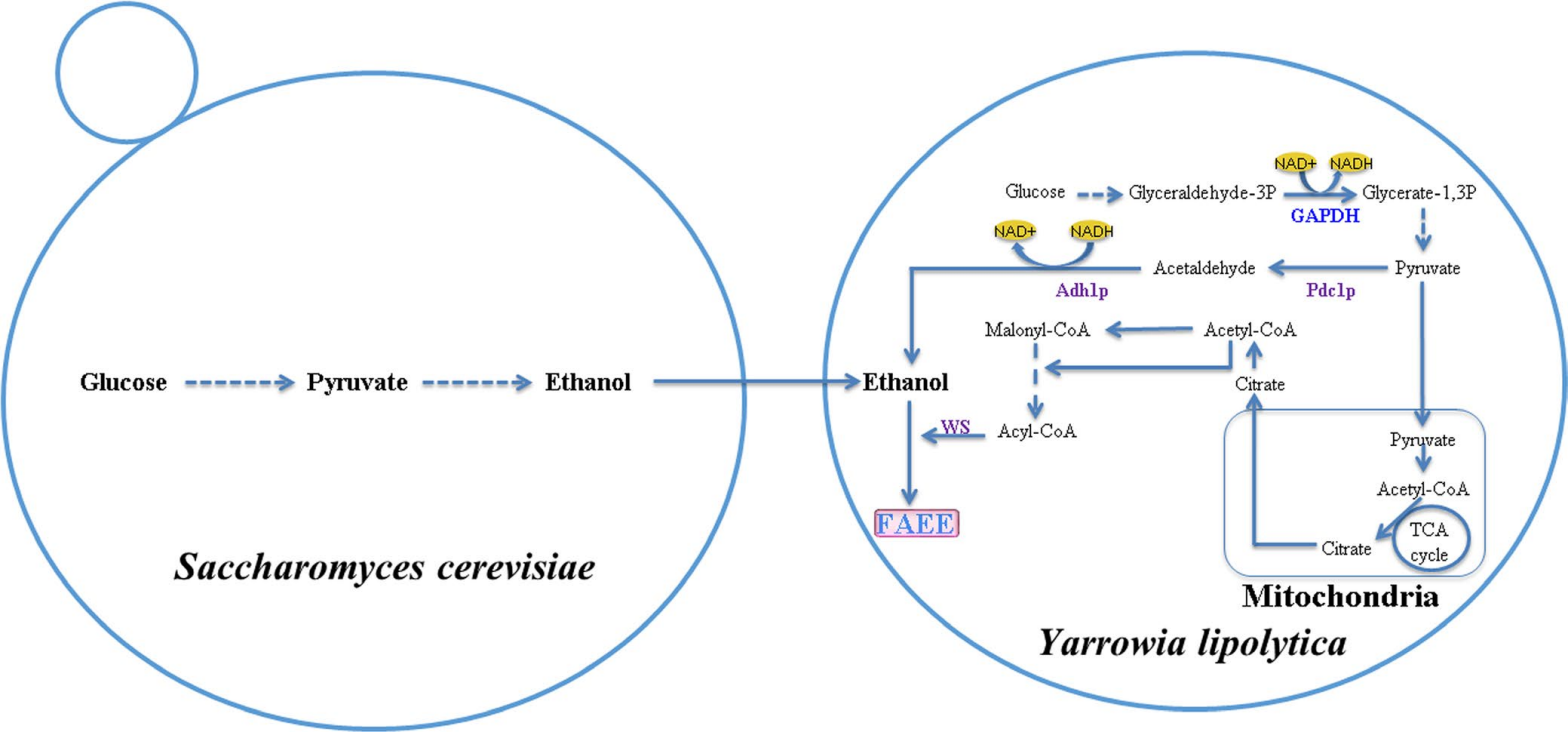
Schematic diagram summarizing metabolic engineering strategies for fatty acid ethyl esters (FAEE) production in engineered *Yarrowia lipolytica* using monoculture and coculture cultivation. The reconstructed biosynthesis pathway for endogenous ethanol production was constructed in *Y. lipolytica* Po1g via co‐overexpression of two heterologous enzymes pyruvate decarboxylase (Pdc1p) and alcohol dehydrogenase (Adh1p) from *Saccharomyces cerevisiae* S288C, one native glyceraldehyde‐3‐phosphate dehydrogenase (GAPDH) from *Y. lipolytica* Po1g for NADH regeneration. Wax ester synthases from *Marinobacter aquaeolei* VT8 (MaWS1) and *Marinobacter hydrocarbonoclasticus* DSM 8798 (MhWS) were then introduced into *Y. lipolytica*, respectively, for FAEE production in the monoculture of *Y. lipolytica*. Homologous and heterologous enzymes are shown in blue and purple, respectively. Solid lines indicate the single‐step reactions of FAEE synthesis in *Y. lipolytica,* and the multi‐step reaction is shown with the dashed line. The FAEE titers in the engineered *Y. lipolytica* strains could be further improved when cocultured with the yeast *S. cerevisiae* that could provide ethanol as a substrate for FAEE production

Xu, Qiao, Ahn, and Stephanopoulos (2016) demonstrated the construction of an engineered *Y. lipolytica* strain for FAEE production through the expression of *A. baylyi* ADP1 targeted to the endoplasmic reticulum and peroxisome. To our knowledge, this was the first report of FAEE production in *Y. lipolytica* using metabolic engineering strategies. Gao et al. (2018) further optimized FAEE production in *Y. lipolytica* through extensive metabolic engineering, and increased FAEE titer was achieved in *Y. lipolytica* with the addition of exogenous ethanol. Very recently, Ng et al. (2020) demonstrated the production of FAEEs from vegetable cooking oil as a model food waste in the engineered *Y. lipolytica*. In this small number of studies conducted to date, the FAEE titers obtained with engineered *Y. lipolytica* strains were low. In this work, we aimed to test the usefulness of new strategies by optimizing FAEE production from endogenously produced ethanol in *Y. lipolytica* and by cocultivation of an ethanol producer with *Y. lipolytica*. Finally, engineered *Y. lipolytica* strain was shown to be capable of producing FAEEs using endogenously produced ethanol. As shown in Figure 1, FAEEs can be generated from ethanol and fatty acyl‐CoA by expressing heterologous WS in our engineered yeast strains. Besides, we demonstrated that a coculture system consisting of the yeasts *Y. lipolytica* and *S. cerevisiae* has potential applications in the sustainable production of FAEEs.

## 2 MATERIALS AND METHODS

### 2.1 Strains, media, and culture conditions

The *E. coli* strain TOP10 was used as the host in this study for the cloning and propagation of plasmids. *E. coli* strains carrying recombinant plasmids were routinely cultured at 37°C in Luria–Bertani (LB) broth containing 1% tryptone, 0.5% yeast extract, and 1% sodium chloride or on LB agar plates supplemented with 100 μg/ml ampicillin. The *Y. lipolytica* strain Po1g, a leucine‐auxotrophic derivative of the wild‐type strain W29 (ATCC 20460), was chosen as a host organism for heterologous gene expression in this study. A yeast coculture of the *S. cerevisiae* strain S288C and the engineered *Y. lipolytica* strain was designed and developed in this study for the direct production of FAEEs. Routine cultivation of the *Y. lipolytica* strains, *S. cerevisiae* strains, and yeast coculture was carried out at 30°C in yeast extract–peptone–dextrose (YPD) liquid medium containing 1% yeast extract, 2% peptone, and 2% dextrose or on YPD agar plates. Synthetic complete medium lacking leucine (YNBleu) and containing 2% glucose and 0.67% yeast nitrogen base w/o amino acids was used for the selection of *Y. lipolytica* Leu^+^ transformants. The strains and plasmids used in this study are listed in Table 1.

**TABLE 1:** Sources and characteristics of strains and plasmids

Strain or plasmid	Relevant properties or genotype	Source
Strains		
*Yarrowia lipolytica* Po1g	*MatA, leu2‐270, ura3‐302::URA3, xpr2‐332, axp‐2 *	Madzak, Tréton, and Blanchin‐Roland (2000)
*Saccharomyces cerevisiae* S288C	*MATα, SUC2, gal2, mal2, mel, flo1, flo8‐1, hap1, ho, bio1, bio6 *	Mortimer and Johnston (1986)
*Escherichia coli* TOP10	*F^‐^mcrA, ∆(mrr‐hsdRMS‐mcrBC), Φ80lacZ ∆M15, ∆lacX74, recA1, araD139, ∆(ara‐leu)7697, galU, galK, rpsL, endA1, nupG*	Invitrogen
Plasmids		
pYLEX1	hp4d promoter, *XPR2* terminator, *LEU2* marker, inserted into the genome at the pBR docking platform of Po1g strain.	Yeastern Biotech
pYLEX1PDC1	pYLEX1 carrying *S. cerevisiae* S288C pyruvate decarboxylase gene *PDC1*	This study
pYLEX1ADH1	pYLEX1 carrying *S. cerevisiae* S288C alcohol dehydrogenase gene *ADH1*	This study
pYLPDC1ADH1	pYLEX1 carrying *PDC1* and *ADH1*	This study
pYLEX1GAPDH	pYLEX1 carrying *Y. lipolytica* Po1g glyceraldehyde*‐*3‐phosphate dehydrogenase gene *GAPDH*	This study
pYLP1A1GA	pYLEX1 carrying *PDC1*, *ADH1* and *GAPDH*	This study
pYLEX1MaAtfA	pYLEX1 carrying *M. aquaeolei* VT8 wax ester synthase gene *MaAtfA*	This study
pYLEX1MhAtfA	pYLEX1 carrying *M. hydrocarbonoclasticus* DSM 8798 wax ester synthase gene *MhAtfA*	This study
pYLP1A1GAMa	pYLEX1 carrying *PDC1*, *ADH1*, *GAPDH* and *MaAtfA*	This study
pYLP1A1GAMh	pYLEX1 carrying *PDC1*, *ADH1*, *GAPDH* and *MhAtfA*	This study

### 2.2 DNA manipulation

**FIGURE A1:**
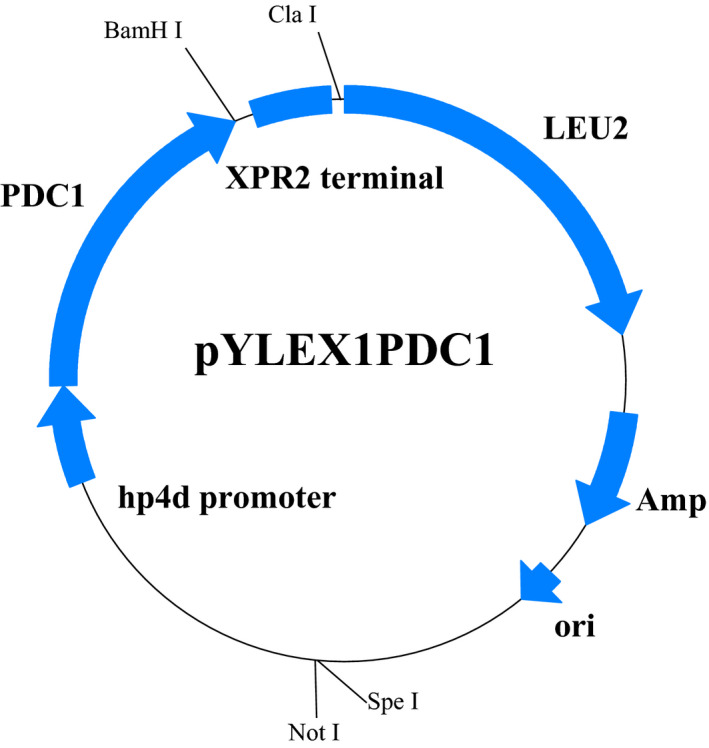
Map of the plasmid pYLEX1PDC1

**FIGURE A2:**
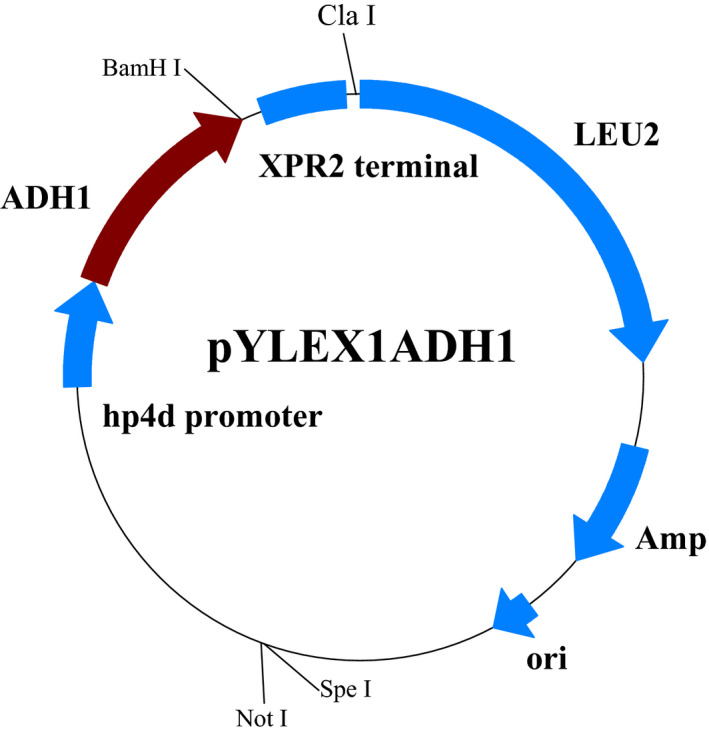
Map of the plasmid pYLEX1ADH1

**FIGURE A3:**
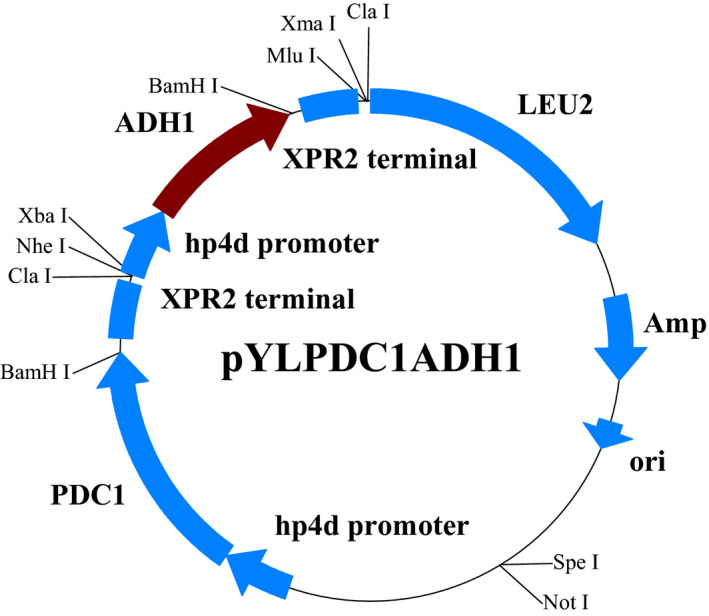
Map of the plasmid pYLPDC1ADH1

**FIGURE A4:**
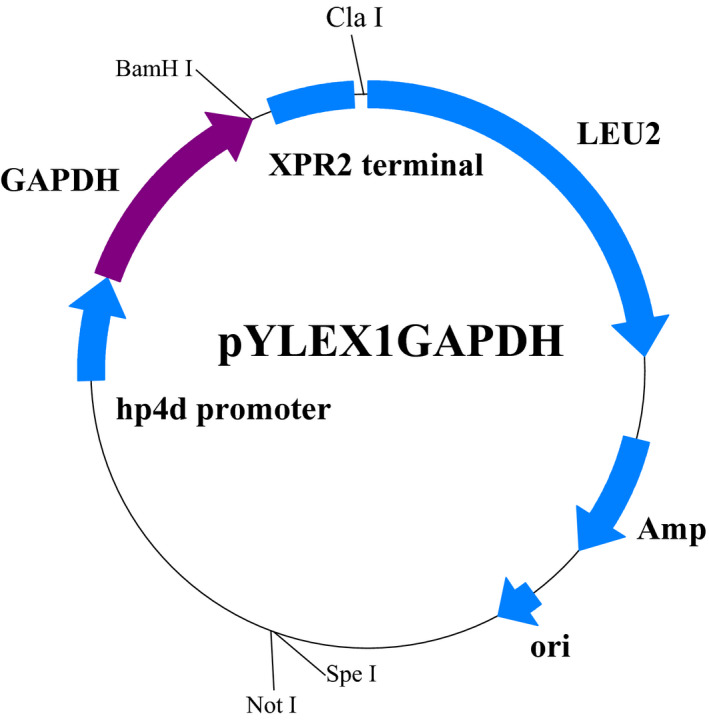
Map of the plasmid pYLEX1GAPDH

**FIGURE A5:**
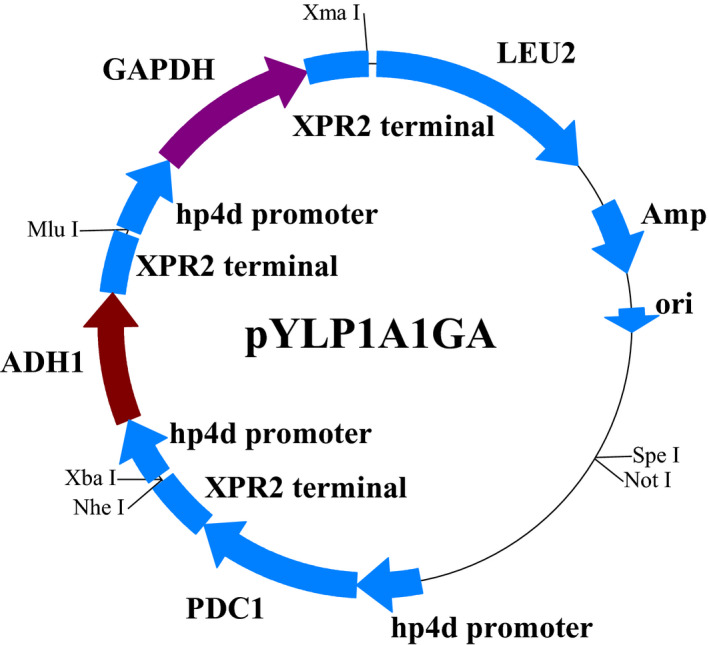
Map of the plasmid pYLP1A1GA

**FIGURE A6:**
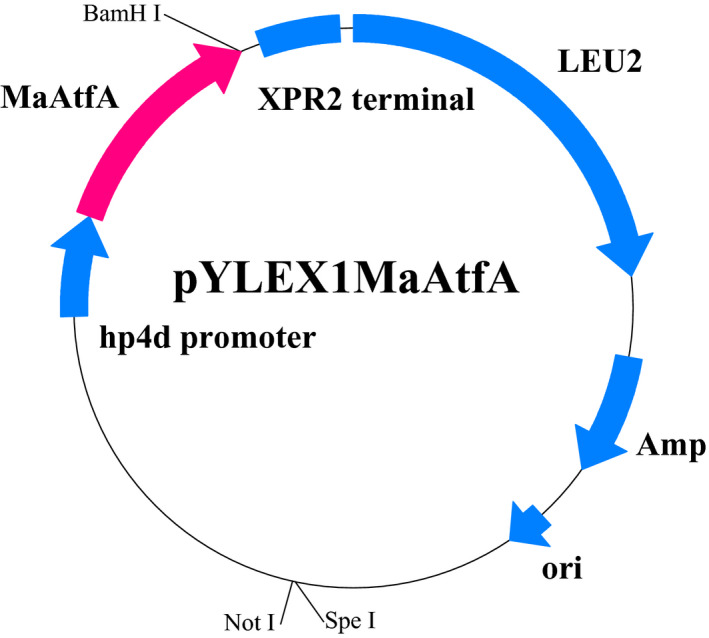
Map of the plasmid pYLEX1MaAtfA

**FIGURE A7:**
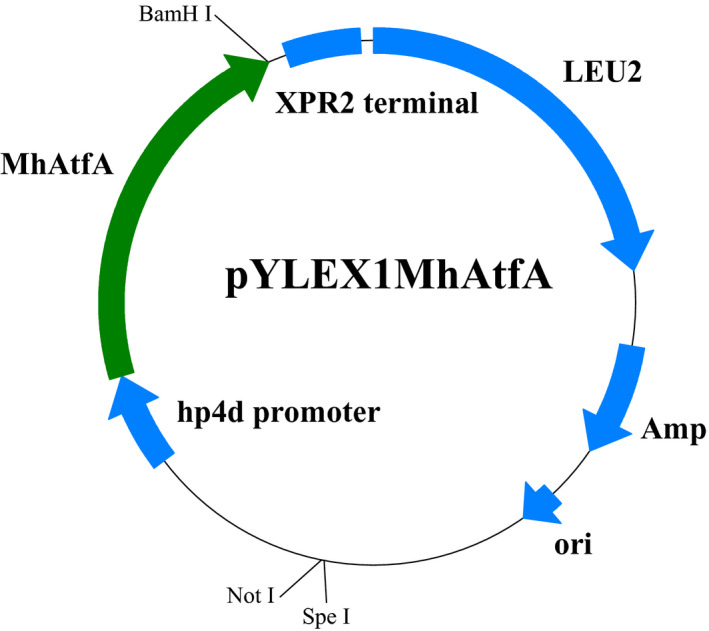
Map of the plasmid pYLEX1MhAtfA

**FIGURE A8:**
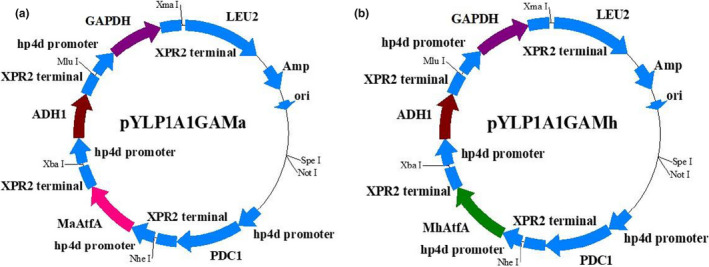
Representative maps of the plasmid vectors containing gene integration cassettes for expression in *Yarrowia lipolytica*. The recombinant vector pYLP1A1GAMa (A) which carries genes *PDC1*, *ADH1*, *GAPDH*, and *MaAtfA*, and the recombinant vector pYLP1A1GAMh (B) which carries genes *PDC1*, *ADH1*, *GAPDH*, and *MhAtfA* were constructed through multiple rounds of restriction digestion and ligation for overexpressing the entire pathway of FAEE synthesis, respectively

The plasmid pYLEX1 (Yeastern Biotech, Taipei, Taiwan) containing a strong hybrid promoter (hp4d) was used for gene expression in the *Y. lipolytica* host strain Po1g. Recombinant plasmids containing different gene expression cassettes were constructed by the following procedures and are graphically depicted in Appendix 1, Figures A1–A8. The *PDC1* and *ADH1* genes from *S. cerevisiae* S288C were ligated into the *Pml* I/*Bam*H I sites of pYLEX1 to yield the plasmids pYLEX1PDC1 and pYLEX1ADH1, respectively (Appendix 1, Figures A1 and A2). The gene expression cassette of *ADH1* was amplified by PCR from pYLEX1ADH1 using a forward primer containing *Cla* I, *Nhe* I, and *Xba* I sites and a reverse primer containing *Cla* I, *Xma* I, and *Mlu* I sites, and then ligated into the *Cla* I site of pYLEX1PDC1 to yield the plasmid pYLPDC1ADH1 (Appendix 1, Figure A3). The native *GAPDH* gene encoding glyceraldehyde‐3‐phosphate dehydrogenase from *Y. lipolytica* Po1g was ligated into the *Pml* I/*Bam*H I sites of pYLEX1 to yield the plasmid pYLEX1GAPDH (Appendix 1, Figure A4). The gene expression cassette of *GAPDH* was amplified by PCR from pYLEX1GAPDH using a forward primer containing a *Mlu* I site and a reverse primer containing a *Xma* I site and then ligated into the *Pml* I/*Bam*H I sites of pYLPDC1ADH1 to yield the plasmid pYLP1A1GA (Appendix 1, Figure A5). Following the construction of pYLP1A1GA, the wax ester synthase genes *MaAtfA* from *M. aquaeolei* VT8 (encoding MaWS1) and *MhAtfA* from *M. hydrocarbonoclasticus* DSM 8798 (encoding MhWS) were ligated into the *Pml* I/*Bam*H I sites of pYLEX1 to yield the plasmids pYLEX1MaAtfA and pYLEX1MhAtfA, respectively (Appendix 1: Figures A6 and A7). The gene expression cassettes of *MaAtfA* and *MhAtfA* were amplified by PCR from pYLEX1MaAtfA and pYLEX1MhAtfA and then ligated into the *Nhe* I/*Xba* I sites of pYLP1A1GA to yield the plasmids pYLP1A1GAMa and pYLP1A1GAMh (Appendix 1, Figure A8). The primers used for gene cloning and plasmid construction are listed in Appendix 1, Table A1.

### 2.3 Competent cell preparation and transformation of *Y. lipolytica* strains

The plasmids pYLEX1, pYLEX1PDC1, pYLEX1ADH1, pYLPDC1ADH1, pYLP1A1GA, pYLEX1MaAtfA, pYLEX1MhAtfA, pYLP1A1GAMa, and pYLP1A1GAMh were first digested with *Spe* I or *Not* I, and the resulting fragments were then integrated into the genome of *Y. lipolytica* Po1g by a chemical transformation process, using a protocol detailed in Appendix 1. The introduced plasmids were integrated at the *URA3* locus of strain Po1g. After transformation, the positive *Y. lipolytica* transformants were selected on YNBleu plates and subsequently confirmed by genomic DNA PCR analysis. Accordingly, the following engineered *Y. lipolytica* strains were generated through chromosomal integration of the recombinant plasmids in the *Y. lipolytica* Po1g strain: (a) Po1g::pYLEX1 (used as a negative control strain), (b) Po1g::pYLEX1PDC1, (c) Po1g::pYLEX1ADH1, (d) Po1g::pYLPDC1ADH1, (e) Po1g::pYLP1A1GA, (f) Po1g::pYLEX1MaAtfA, (g) Po1g::pYLEX1MhAtfA, (h) Po1g::pYLP1A1GAMa, and (i) Po1g::pYLP1A1GAMh. Five consecutive passages were conducted to evaluate the genetic stability of the genetically engineered *Y. lipolytica* strains. Subsequently, each strain was subjected to gas chromatography–mass spectrometry (GC/MS) analysis of microbial end products.

### 2.4 GC/MS analysis of FAEEs and ethanol produced in engineered *Y. lipolytica* strains

To measure the production of FAEEs and ethanol in engineered *Y. lipolytica* strains, seed cultures were prepared by inoculating 5 ml of YPD medium in 50‐mL culture tubes with the corresponding strains. The cells were incubated overnight with continuous agitation. Next, 250‐mL flasks containing 50 ml of YPD medium were inoculated with freshly prepared seed cultures to obtain an OD_600_ of 0.05. All cultures were shaken at 225 rpm and 30°C. Samples were then collected at different time points after the start of cultivation, and a 5‐ml sample of each culture was centrifuged. The growth of the engineered yeast strains was examined by measuring the optical density at 600 nm (OD_600_) using a spectrophotometer during the culture period. For the quantification of extracellular ethanol and FAEEs, products were extracted from 2 ml of the culture supernatant (3‐day culture) by vortexing for 2 min with 2 ml of n‐hexane. For the quantification of intracellular ethanol and FAEEs, the cell pellet was lysed by eight rounds of 30‐s bead beating with 1 min of cooling on ice between each round. Following cell lysis, products were extracted by vortexing for 2 min with 2 ml of n‐hexane. The n‐hexane extracts were then analyzed by GC/MS using an HP 7890B GC with an Agilent 5977A MSD equipped with an HP‐FFAP capillary column (Agilent Technologies, Wilmington, USA). The GC oven temperature was initially held at 50°C for 1 min and then ramped to 210°C at a rate of 10°C/min and held for 5 min. The temperature was subsequently ramped at 5°C/min to 280°C and held for 5 min. Helium was used as the carrier gas, with an inlet pressure of 13.8 psi. The injector was maintained at 280°C, and the ion source temperature was set to 230°C. FAEE levels were quantified by comparing the integrated peak area of the samples with those of the corresponding standards. Final data analysis was performed using Enhanced Data Analysis software (Agilent, USA).

## 3 RESULTS AND DISCUSSION

### 3.1 Construction and characterization of an ethanol synthetic pathway in *Y. lipolytica*

**FIGURE 2:**
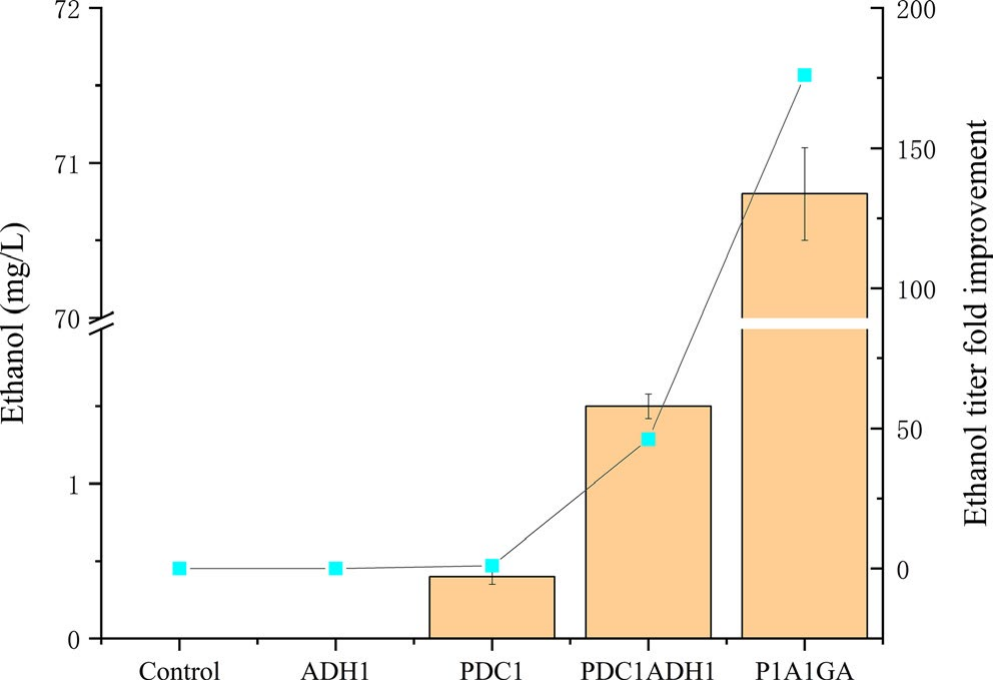
Production of ethanol by metabolically engineered strains of *Yarrowia lipolytica* in shake flasks. All the *Y. lipolytica* strains were cultivated aerobically at 30°C, with vigorous shaking in yeast extract‐peptone‐dextrose medium, and ethanol titers were determined using GC/MS at the 72‐hr time point. Bars represent the corresponding titers attained by different *Y. lipolytica* strains, and lines represent ethanol titer improvement over PDC1. Control, ADH1, PDC1, PDC1ADH1, and P1A1GA refer to *Y. lipolytica* Po1g strain carrying integrated plasmids pYLEX1 (empty vector), pYLEX1ADH1, pYLEX1PDC1, pYLPDC1ADH1, and pYLP1A1GA, respectively. All titer values shown represent the averages ± standard deviations of the results of three independent biological replicates

In microorganisms, the production of FAEEs requires two substrates: ethanol and fatty acyl‐CoA (or free fatty acids). Unlike the conventional yeast *S. cerevisiae*, the unconventional yeast *Y. lipolytica* does not produce significant ethanol levels (Barth & Gaillardin, 1996). Therefore, to construct a complete pathway for the formation of ethanol from pyruvate in *Y. lipolytica*, the two genes *PDC1* (encoding pyruvate decarboxylase) and *ADH1* (encoding alcohol dehydrogenase), which are involved in the ethanol biosynthetic pathway of *S. cerevisiae,* were selected and subsequently introduced into *Y. lipolytica* (Figure 1). The engineered *Y. lipolytica* strain coexpressing the *S. cerevisiae PDC1* and *ADH1* genes was then tested for ethanol production from glucose. The resulting engineered strain Po1g::pYLEX1‐PDC1ADH1 produced ethanol at a concentration of 1.5 mg/L after 72 hr of cultivation, and no ethanol was detected in the negative control strain Po1g::pYLEX1, demonstrating that the heterologous enzymes encoded by the *PDC1* and *ADH1* genes that had been integrated into the genome of *Y. lipolytica* could fulfill their functions in *Y. lipolytica*. No ethanol was detected in the Po1g::pYLEX1‐ADH1 strain, and the ethanol titer accumulated in the engineered Po1g::pYLEX1‐PDC1 strain (also used as a control) was only 0.4 mg/L (Figure 2). This finding suggests that pyruvate decarboxylases of *Y. lipolytica* do not have enough activity, carbon flux to the TCA cycle outcompetes PDC activity, or PDC expression is downregulated in glucose medium. Because *S. cerevisiae* Adh1p catalyzes the reduction of acetaldehyde to ethanol using NADH as the enzyme cofactor, it is proposed that by overexpressing oxidative enzymes that utilize NAD^+^ as the cofactor, regeneration of NADH can be accelerated, thus providing sufficient supply of NADH for the ethanol–acetaldehyde shuttle, which, in turn, will improve the ability of *Y. lipolytica* to synthesize ethanol. To this end, the native *GAPDH* gene encoding a glyceraldehyde‐3‐phosphate dehydrogenase that oxidizes glyceraldehyde‐3‐phosphate to 1,3‐bisphosphoglycerate in *Y. lipolytica *(Linck et al., 2014), was chosen for overexpression (Figure 1) because we had previously demonstrated that overexpression of *GAPDH* can effectively recycle the NADH required for 1‐butanol biosynthesis and thus boost 1‐butanol production in *Y. lipolytica *(Yu et al., 2018). The titer of ethanol in the resulting engineered strain Po1g::pYLP1A1GA after 72 hr of cultivation increased markedly to 70.8 mg/L, which is a 176.0‐fold increase compared to the titer in the Po1g::pYLEX1‐PDC1 strain and a 46.2‐fold increase compared to the titer in the Po1g::pYLEX1‐PDC1ADH1 strain (Figure 2), and this increase had no significant effects on the cell growth of *Y. lipolytica*. Thus, our results revealed that the NADH cofactor regeneration strategy could effectively improve ethanol biosynthesis in *Y. lipolytica*. Interestingly, it was found that ethanol accumulated mainly intracellularly in all of the engineered strains and the extracellular ethanol titer was less than 1 mg/L (at the μg/L level). A possible reason for such a situation is that the resulting ethanol was trapped intracellularly due to the relatively low level of ethanol production. To our knowledge, this is the first report on the successful production of ethanol in the non‐ethanol‐producing yeast *Y. lipolytica*. However, the ethanol titers obtained in the engineered *Y. lipolytica* strains are quite low compared to the production of ethanol in the ethanol‐producing yeast *S. cerevisiae *(Najafpour, Younesi, & Ismail, 2004). Therefore, this heterologous ethanol biosynthetic pathway and the genes that we selected require further optimization to boost the titers.

### 3.2 Integration of the *atfA *gene, encoding a wax ester synthase, in *Y. lipolytica*

**FIGURE 3:**
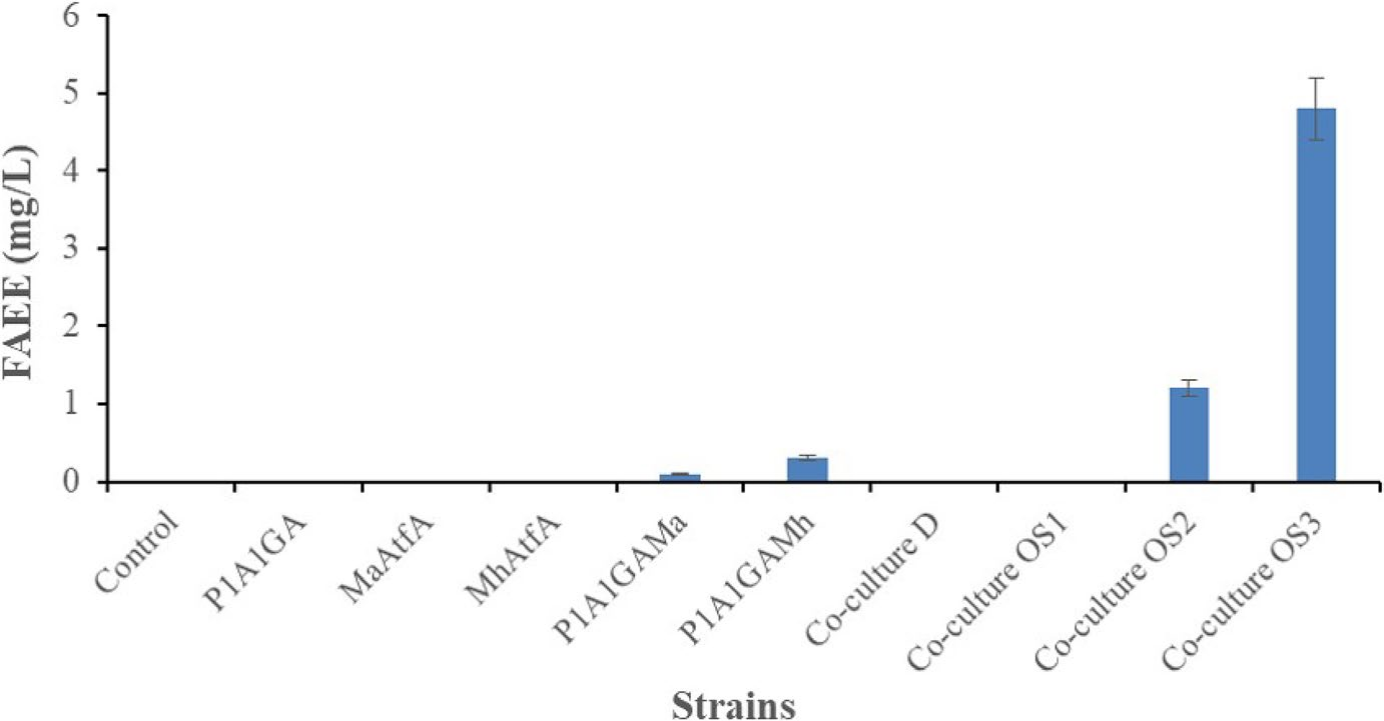
Production of fatty acid ethyl esters (FAEEs) by the engineered strains of *Yarrowia lipolytica* and the *Y. lipolytica*–*Sacharomyces cerevisiae* coculture. All the yeast strains were cultivated aerobically at 30°C, with vigorous shaking in the yeast extract‐peptone‐dextrose medium. The FAEE titers achieved from the corresponding *Y. lipolytica* strains were analyzed using GC/MS after 3 days of cultivation, and the FAEE titers achieved from the coculture were analyzed at different time points after the start of cultivation as described in Section 3.3. Control, P1A1GA, MaAtfA, MhAtfA, P1A1GAMa, and P1A1GAMh refer to *Y. lipolytica* Po1g strain carrying integrated plasmids pYLEX1 (empty vector), pYLP1A1GA, pYLEX1MaAtfA, pYLEX1MhAtfA, pYLP1A1GAMa, and pYLP1A1GAMh, respectively. Co‐culture D, Co‐culture OS1, Co‐culture OS2, and Co‐culture OS3 represent the microbial coculture under the conditions of the original coculture strategy, the first coculture optimization strategy, the second coculture optimization strategy, and the third coculture optimization strategy, respectively, as described in Section 3.3. All titer values shown represent the averages ± standard deviations of the results of three independent biological replicates

**FIGURE 4:**
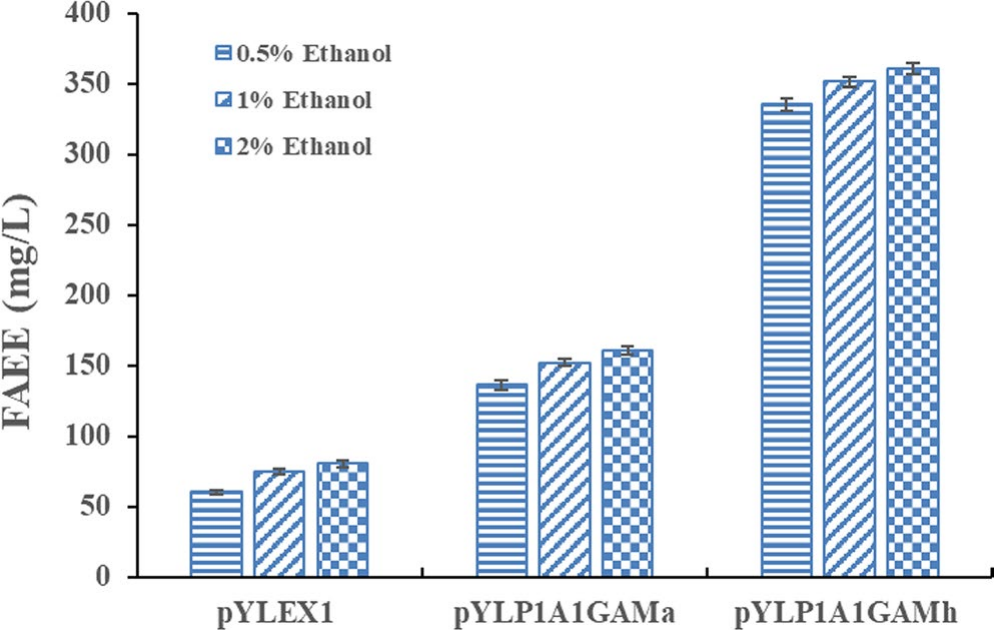
Fatty acid ethyl esters (FAEE) production in engineered *Yarrowia lipolytica* strains expressing wax ester synthases MaWS1 or MhWS supplemented with different concentrations of ethanol. All *Y. lipolytica* strains were cultivated in YPD medium supplemented with different concentrations (0.5%, 1%, 2%) of ethanol. FAEE titers achieved from the corresponding strains were analyzed using GC/MS after 3 days of cultivation. All titer values shown represent the averages ± standard deviations of the results of three independent biological replicates

It has been reported that several wax ester synthases can efficiently catalyze the esterification of acyl‐CoA to ethanol to synthesize FAEEs due to their nonspecificity (Barney et al., 2012; Kalscheuer et al., 2006; Steen et al., 2010; Wierzbicki et al., 2016). Thus, we propose that overexpression of wax ester synthase genes in *Y. lipolytica* could potentially permit the engineered *Y. lipolytica* strains to increase FAEE production. With the successful construction of ethanol biosynthetic pathways in *Y. lipolytica*, the esterification enzyme activity that links fatty acyl‐CoA (or fatty acid) to ethanol is required to enable FAEE synthesis in *Y. lipolytica* (Figure 1). Herein, two wax ester synthases were selected because the heterologous expression of each candidate gene (*MaAtfA* or *MhAtfA*) has previously shown great potential for the production of FAEEs in multiple host strains as described in the Introduction section (Barney et al., 2012; Shi et al., 2012; Teo, Ling, Yu, & Chang, 2015). The growth profiles of both engineered strains were similar to that of the parental strain, indicating that integration of multiple expressible heterologous genes into the chromosome of *Y. lipolytica* did not have any adverse effect on cell growth. After 72 hr of cultivation, FAEEs were produced at a titer of 0.1 and 0.3 mg/L in the Po1g::pYLP1A1GAMa strain and Po1g::pYLP1A1GAMh strain, respectively. FAEEs were not detected in any of the remaining seven strains, namely, Po1g::pYLEX1, Po1g::pYLEX1PDC1, Po1g::pYLEX1ADH1, Po1g::pYLPDC1ADH1, Po1g::pYLP1A1GA, Po1g::pYLEX1MaAtfA, and Po1g::pYLEX1MhAtfA (Figure 3). However, the highest titer obtained in FAEE‐producing *Y. lipolytica* strain (Po1g::pYLP1A1GAMh) remained rather low compared to similar examples in the literature on FAEE production in either *E. coli* or *S. cerevisiae *(Kalscheuer et al., 2006; Shi et al., 2012). One reason for this discrepancy may be that heterologous expression of either *MaAtfA* or *MhAtfA* did not confer adequate FAEE biosynthetic ability to the *Y. lipolytica* host strain despite the presence of two substrates. Another reason is that the supply of ethanol in the engineered *Y. lipolytica* strains remained insufficient to meet the demands for high‐level biosynthesis of FAEEs as described above. To verify this hypothesis, the effect of exogenous ethanol was evaluated on the potential for FAEE production in the engineered *Y. lipolytica* strains. The results showed that FAEE production by the engineered *Y. lipolytica* strains was greatly enhanced by the addition of exogenous ethanol, as expected (Figure 4). In the presence of 0.5%, 1%, and 2% (v/v) exogenous ethanol, the Po1g::pYLP1A1GAMa strain achieved total FAEE titers of 136.4, 152.1, and 160.4 mg/L, respectively, after 72 hr of cultivation, while *MhAtfA* overexpression led to higher total FAEE titers (335.8, 351.4, 360.8 mg/L) in the Po1g::pYLP1A1GAMh strain than in the Po1g::pYLP1A1GAMa strain. Interestingly, total FAEE titers of 60.2, 74.8, and 80.3 mg/L (in the presence of 0.5%, 1% and 2% (v/v) exogenous ethanol, respectively) were also observed in the Po1g::pYLEX1 strain which was used as the control strain. This result can be attributed to the esterification activity of endogenous lipases in *Y. lipolytica* that can catalyze the production of FAEEs (Cao et al., 2017; Matsumoto, Ito, Fukuda, & Kondo, 2004; Meng et al., 2011).

A large increase in the FAEE yield was demonstrated for the Po1g::pYLP1A1GAMa strain and the Po1g::pYLP1A1GAMh strain in the presence of exogenous ethanol as compared to the Po1g::pYLEX1 strain and strains grown in the absence of ethanol. This result indicated that (a) both heterologous enzymes (MaWS1 and MhWS) selected herein could fulfill the roles in catalyzing the formation of FAEEs in *Y. lipolytica* and that (b) increasing ethanol concentration could boost the production of FAEEs. A higher ethanol concentration was not used because the growth of the corresponding *Y. lipolytica* strains was greatly affected when the concentration of exogenous ethanol was increased to 3%. The Po1g::pYLP1A1GAMh strain with the highest level of FAEE production (which was 3.8‐fold higher than that of the Po1g::pYLEX1 strain) and with a 1.2‐fold increase in FAEE titer over the Po1g::pYLP1A1GAMa strain when fed with 2% ethanol was therefore used for subsequent work in this study. The total FAEEs produced by the Po1g::pYLP1A1GAMh strain at an ethanol concentration of 2% contained 10.9% butanoic acid ethyl ester (C4:0), 0.4% decanoic acid ethyl ester (C10:0), 1.2% dodecanoic acid ethyl ester (C12:0), 1.9% myristic acid ethyl ester (C14:0), 56.4% palmitic acid ethyl ester (C16:0), 5.6% palmitoleic acid ethyl ester (C16:1n‐7), 20.2% stearic acid ethyl ester (C18:0), 2.3% oleic acid ethyl ester (C18:1n‐9), and 1.1% linoleic acid ethyl ester (C18:2n‐6), of which palmitic acid ethyl ester, with a sixteen‐carbon saturated fatty acid chain, accounted for more than half of the total FAEE yield. Furthermore, and interestingly, butanoic acid ethyl ester, with a short‐chain fatty acid, accounted for more than 10% of the total yield (Table 2). The phenotype of the Po1g::pYLP1A1GAMh strain was considerably stable even after five consecutive passages, indicating high genetic stability.

**TABLE 2:** The percentage composition of total FAEEs produced by *Yarrowia lipolytica* strains under different culture conditions

Product	Sample 1 (%)	Sample 2 (%)	Sample 3 (%)
Butanoic acid ethyl ester (C4:0)	10.9	8.6	BD
Decanoic acid ethyl ester (C10:0)	0.4	BD	0.1
Dodecanoic acid ethyl ester (C12:0)	1.2	BD	0.3
Myristic acid ethyl ester (C14:0)	1.9	BD	0.4
Palmitic acid ethyl ester (C16:0)	56.4	51.7	25.7
Palmitoleic acid ethyl ester (C16:1n*‐*7)	5.6	2.6	14.1
Stearic acid ethyl ester (C18:0)	20.2	18.1	2.9
Oleic acid ethyl ester (C18:1n*‐*9)	2.3	14.6	37.8
Linoleic acid ethyl ester (C18:2n*‐*6)	1.1	4.4	18.7

Note

FAEEs produced in shake flasks with YPD media were separated and quantified by GC/MS. All values presented are the mean of three biological replicates. Sample 1 represents the Po1g::pYLP1A1GAMh strain fed with a concentration of 2% ethanol as described in Section 3.2. Sample 2 and Sample 3 represent the microbial coculture under the conditions of the second and the third coculture optimization strategies, respectively, as described in Section 3.3. BD represents “below the detection level.”

### 3.3 FAEE production by cocultivation of the *Y. lipolytica* Po1g::pYLP1A1GAMh strain and *S. cerevisiae* S288C strain

As described above, the highest titer of FAEEs achieved in the engineered *Y. lipolytica* strain was 360.8 mg/L. This titer was much higher than that achieved in the yeast *S. cerevisiae* harboring the same wax ester synthase from *M. hydrocarbonoclasticus* DSM 8798 (6.3 mg/L) (Shi et al., 2012). Based on the results obtained here, *Y. lipolytica* is a more promising candidate yeast species for future applications in FAEE production than *S. cerevisiae*.

However, ethanol insufficiency is a major bottleneck in the development of *Y. lipolytica* as a high‐level FAEE producer, and supplying additional ethanol to the culture medium of *Y. lipolytica* can boost product levels of FAEEs. This finding was confirmed by the results of FAEE production in the Po1g::pYLP1A1GAMh strain when exogenous ethanol was added into the culture medium. Thus, a deeper understanding of the flux throughout the ethanol biosynthetic route in *Y. lipolytica* is necessary to identify and eliminate the bottlenecks and/or possible competing pathways and, in turn, further improve ethanol accumulation in this oleaginous yeast system.

Instead of further boosting ethanol production in *Y. lipolytica* through metabolic engineering, which might be a very difficult task, in the present study, we proposed the cocultivation of *S. cerevisiae* and *Y. lipolytica* to produce FAEEs in *Y. lipolytica* using ethanol provided by *S. cerevisiae*. Many studies have reported the use of microbial coculture systems for direct production of specific target compounds, increasing the production yield, shortening the fermentation time, and/or reducing the process cost and/or realizing some specific function (He, Duan, & Liu, 2014; Hickert, Cunha‐Pereira, Souza‐Cruz, Rosa, & Ayub, 2013; Minty et al., 2013; Singh, Bajar, & Bishnoi, 2014; Zhou, Qiao, Edgar, & Stephanopoulos, 2015). We therefore sought to investigate whether the use of a microbial coculture system could successfully contribute to the production of FAEEs and potentially enhance FAEE fermentation performance by coculturing the *S. cerevisiae* S288C strain with the engineered *Y. lipolytica* strain Po1g::pYLP1A1GAMh, since *S. cerevisiae* can metabolize glucose to produce relatively large amounts of ethanol.

To this end, the effect of different coculture designs on FAEE production was investigated using shake‐flask experiments. When seed cultures of *S. cerevisiae* S288C and *Y. lipolytica* Po1g::pYLP1A1GAMh with the same initial OD_600_ of 0.05 were simultaneously inoculated into one flask containing YPD medium (the original coculture strategy), we noted that the production yield of FAEEs in the coculture samples was below the detection limit after three days of cultivation, suggesting that this coculture condition is unsuitable for FAEE accumulation in this original mixed microbial culture. Another cause for this effect could be the unsuitable condition for FAEE accumulation in this original mixed microbial culture.

To validate the hypothesis, FAEE production under different coculture conditions was investigated next. The first coculture optimization strategy was as follows: *S. cerevisiae* S288C was first cultured separately for 24 hr, and then, a fresh overnight seed culture of *Y. lipolytica* Po1g::pYLP1A1GAMh was inoculated at an initial OD_600_ of 0.2 into the fermentation medium of *S. cerevisiae*. The coculture sample was collected after 3 days for subsequent GC/MS analysis. Under the conditions of the first coculture strategy, GC/MS analysis results also indicated that FAEEs were not present in the mixed coculture performed by sequential inoculation of *S. cerevisiae* and *Y. lipolytica*. The second coculture optimization strategy was as follows: *Y. lipolytica* Po1g::pYLP1A1GAMh was first cultured for 24 hr, and then, fresh overnight seed culture of *S. cerevisiae* S288C was inoculated at an initial OD_600_ of 0.2 into the fermentation medium of *Y. lipolytica*. The coculture sample was collected after 2 days for subsequent GC/MS analysis. Under the conditions of the second coculture strategy, the synthetic consortium produced FAEEs at a titer of 1.2 mg/L (Figure 3). The total FAEEs contained 8.6% butanoic acid ethyl ester (C4:0), 51.7% palmitic acid ethyl ester (C16:0), 2.6% palmitoleic acid ethyl ester (C16:1n‐7), 18.1% stearic acid ethyl ester (C18:0), 14.6% oleic acid ethyl ester (C18:1n‐9), and 4.4% linoleic acid ethyl ester (C18:2n‐6). Also, the concentrations of decanoic acid ethyl ester, dodecanoic acid ethyl ester, and myristic acid ethyl ester decreased to below the detection limit, unlike the concentrations in *Y. lipolytica* (Po1g::pYLP1A1GAMh strain) alone (Table 2). The third coculture optimization strategy was as follows: *S. cerevisiae* S288C and *Y. lipolytica* Po1g::pYLP1A1GAMh were cultured separately for 24 hr with continuous shaking in YPD medium, and then, 25 ml of a 24 hr inoculum of each was transferred into a new flask and incubated for an additional 48 hr. Under the conditions of the third coculture strategy, this synthetic consortium produced FAEEs at a titer of 4.8 mg/L (Figure 3). This titer represents a 3.0‐fold increase compared to the second coculture strategy, which indicates that the third coculture strategy provides the best conditions for the production of FAEEs among the three strategies tested in the present study for the current coculture system. Under this condition, the total FAEEs contained 0.1% decanoic acid ethyl ester (C10:0), 0.3% dodecanoic acid ethyl ester (C12:0), 0.4% myristic acid ethyl ester (C14:0), 25.7% palmitic acid ethyl ester (C16:0), 14.1% palmitoleic acid ethyl ester (C16:1n‐7), 2.9% stearic acid ethyl ester (C18:0), 37.8% oleic acid ethyl ester (C18:1n‐9), and 18.7% linoleic acid ethyl ester (C18:2n‐6) (Table 2). The composition of the FAEEs produced by the *Y. lipolytica*–*S. cerevisiae* coculture under this condition was quite different from that in *Y. lipolytica* (Po1g::pYLP1A1GAMh strain) cultured alone at an ethanol concentration of 2% and that obtained from the second coculture optimization strategy. In particular, the concentrations of palmitic acid ethyl ester (C16:0) and stearic acid ethyl ester (C18:1n‐9) decreased significantly, while the concentrations of palmitoleic acid ethyl ester, oleic acid ethyl ester, and linoleic acid ethyl ester, with unsaturated carbon chains, increased substantially. Additionally, the FAEE composition of this mixed microbial culture was dominated by carbon chain lengths of C16 and C18 without detectable amounts of butanoic acid ethyl ester.

Interestingly, the FAEE titer obtained from the two‐yeast‐strain coculture system is lower than that of the single yeast strain *Y. lipolytica* Po1g::pYLP1A1GAMh fed with exogenous ethanol. We therefore measured ethanol concentrations in coculture systems. Results showed that the ethanol concentrations in coculture systems (180.5, 440.8, 500.4 mg/L for the first, second, and third coculture conditions, respectively) are much lower than 1% (v/v) which was produced in the monoculture of *S. cerevisiae*. One of the possible reasons for these results may be that the metabolism of two strains changed when they compete for growth resources in one system (Hettich, Sharma, Chourey, & Giannone, 2012; Khan et al., 2018; Zhou et al., 2015). However, this aspect needs to be investigated further in future work. Based on these results, we confirmed that the application of this microbial coculture system could be a viable strategy for the sustainable production of FAEEs. With the increasing knowledge, it is believed that this coculture system could be further optimized to result in a significant improvement in FAEE production.

## 4 CONCLUSIONS

The nonconventional oleaginous yeast *Y. lipolytica* has previously been shown to be a competitive host organism for the production of fatty acid‐derived products owing to several competitive advantages over other microbial species. In this proof‐of‐principle study, an efficient and eco‐friendly catalytic route for the synthesis of FAEE biodiesel was established in the oleaginous yeast *Y. lipolytica* through metabolic engineering and the coincubation strategy adopted in this study was also shown to be very promising for future FAEE production. Meanwhile, this study also described the successful biosynthesis of ethanol in metabolically engineered *Y. lipolytica*. In conclusion, this work demonstrates the feasibility of adopting *Y. lipolytica* as an engineered cell factory for FAEE production and provides a starting point for advancing the microbe‐based industrial production of FAEE biodiesel, which could be an environmentally friendly and sustainable solution for green fuel (biodiesel) production. However, there remains much room for improvement in the use of *Y. lipolytica* for the production of FAEEs to reach a commercially acceptable level. First, future efforts should focus on screening novel sources of ester synthase enzymes from vastly different organisms with desirable features such as higher enzyme activity, stability, and specificity when expressed in *Y. lipolytica*. Second, various novel metabolic engineering strategies for maximizing ethanol biosynthesis and fatty acyl‐CoA (or free fatty acids) biosynthesis as well as further enhancing coproduction of ethanol and fatty acyl‐CoA in the engineered *Y. lipolytica* strains should be properly designed and applied. Third, strains and culture conditions in the coculture system need to be further optimized to accomplish a marked improvement in the FAEE titer.

## CONFLICT OF INTEREST

None declared.

## AUTHOR CONTRIBUTIONS

**Aiqun Yu:** Conceptualization (lead); investigation (lead); formal analysis (lead); writing – original draft (lead); writing – review and editing (equal); **Yu Zhao, Jian Li, Shenglong Li, Yaru Pang, and Yakun Zhao:** Investigation (supporting); formal analysis (supporting); writing – original draft (supporting); writing – review and editing (equal); **Cuiying Zhang and Dongguang Xiao:** Conceptualization (supporting); writing – original draft (supporting); writing – review and editing (equal).

## Data Availability

All data generated or analyzed during this study are included in this published article and the appendices.

## APPENDIX 1

### Reagents

iProof high‐fidelity DNA polymerase was purchased from Bio‐Rad Labs (Hercules, CA, USA). Restriction enzymes, T4 DNA ligase, Taq DNA polymerase, and PCR reagents were purchased from New England Biolabs (Beverly, MA, USA). CSM‐Leu (complete supplement mixture minus leucine) dropout mixture was purchased from MP Biomedicals (Solon, OH, USA). Yeast extract and peptone were purchased from BD Biosciences (San Jose, CA, USA). Oligonucleotides were synthesized by Takara Biotechnology (Dalian, Liaoning, China). FAEE standards were purchased from Cayman Chemical (Ann Arbor, MI, USA). All other reagents were purchased from Sigma Aldrich (St. Louis, MO, USA) unless otherwise stated.

### Preparation of *Y. lipolytica* Po1g competent cell

1. Inoculate a colony of *Y. lipolytica* Po1g strain from a fresh YPD plate in 10 ml YPD medium (1% yeast extract, 2% peptone, 2% dextrose, and 50 mM citrate buffer pH 4.0) in a 250‐ml flask. Incubate with shaking at 225 rpm at 30°C for 20 hr.
2. Pellet the cells by centrifuging 5 min at 5,000 *g* at room temperature.
3. Wash the cells with 20 ml TE buffer and pellet the cells similarly as Step 2.
4. Resuspend the cells in 1 ml of 0.1 M lithium acetate (pH 6.0, adjusted with acetic acid) and incubate for 10 min at room temperature.
5. Aliquot the competent cells (100 µl) into sterile 1.5‐ml tubes. Proceed to the transformation steps below immediately, or add glycerol to a final concentration of 25% (v/v) and store at −80°C for long‐term storage.

### Transformation of *Y. lipolytica* Po1g cells

1. Gently mix 10 µl of denatured salmon sperm DNA (10 mg/ml) and 1–5 µg of the linearized plasmid with 100 µl of competent cells, and incubate at 30°C for 15 min.
2. Add 700 µl of 40% PEG‐4000 (dissolved in 0.1 M lithium acetate pH 6.0), mix well, and incubate at 30°C for 60 min with shaking (225 rpm).
3. Heat shock the transformation mixture at 39°C for 60 min.
4. Add 1 ml YPD medium and recover for 2 hr at 30°C and 225 rpm.
5. Centrifuge at 10,000 *g* for 1 min, remove supernatant, and resuspend the pellet in 1 ml of TE buffer.
6. Repellet the cells and discard supernatant again.
7. Resuspend the pellet in 100 µl of TE buffer and plate onto selective plates (leucine‐deficient plates).

**TABLE A1:** Primers used to perform heterologous gene expression in this study

Primers	Sequences	Usage
1	5′‐AATGTCTGAAATTACTTTGGG	Cloning *PDC1* gene of *Saccharomyces cerevisiae* S288C into pYLEX1 for the construction of pYLEX1PDC1, forward primer.
2	5′‐CGGGATCCTTATTGCTTAGCGTTGGTAG	Cloning *PDC1* gene of *S. cerevisiae* S288C into pYLEX1 for construction of pYLEX1PDC1, reverse primer.
3	5′‐AATGTCTATCCCAGAAACTCA	Cloning *ADH1* gene of *S. cerevisiae* S288C into pYLEX1 for construction of pYLEX1ADH1, forward primer.
4	5′‐CGGGATCCTTATTTAGAAGTGTCAACAA	Cloning *ADH1* gene of *S. cerevisiae* S288C into pYLEX1 for construction of pYLEX1ADH1, reverse primer.
5	5′‐AATGGCCATCAAAGTCGGTAT	Cloning *GAPDH* gene of *Y. lipolytica* Po1g into pYLEX1 for construction of pYLEX1GAPDH, forward primer.
6	5′‐CGGGATCCCTAAGCGGAAGCATC CTTCT	Cloning *GAPDH* gene of *Y. lipolytica* Po1g into pYLEX1 for construction of pYLEX1GAPDH, reverse primer.
7	5′‐CCATCGATGCTAGCTCTAGAGCTCTCCCTTATGCGACT	Amplifying *ADH1* expression cassette from pYLEX1ADH1 into pYLEX1PDC1 to yield pYLPDC1ADH1, forward primer.
8	5′‐CCATCGATCCCGGGACGCGTGAATTCGGACACGGGCAT	Amplifying *ADH1* expression cassette from pYLEX1ADH1 into pYLEX1PDC1 to yield pYLPDC1ADH1, reverse primer.
9	5′‐CGACGCGTGCTCTCCCTTATGCGACT	Amplifying *GAPDH* expression cassette from pYLEX1GAPDH into pYLPDC1ADH1 to yield pYLP1A1GA, forward primer.
10	5′‐CCCCCGGGGAATTCGGACACGGGCAT	Amplifying *GAPDH* expression cassette from pYLEX1GAPDH into pYLPDC1ADH1 to yield pYLP1A1GA, reverse primer.
11	5′‐AATGACTCCATTGAACCCAAC	Cloning *MaAtfA* gene of *Marinobacter aquaeolei* VT8 into pYLEX1 for construction of pYLEX1MaAtfA, forward primer.
12	5′‐CGGGATCCTCAGTGATGGTGATGATGATGTAAACCAGCGTTCAATTCCA	Cloning *MaAtfA* gene of *M. aquaeolei* VT8 into pYLEX1 for construction of pYLEX1MaAtfA, reverse primer.
13	5′‐AATGAAGAGATTGGGTACTTT	Cloning *MhAtfA* gene of *Marinobacter hydrocarbonoclasticus* DSM 8798 into pYLEX1 for construction of pYLEX1MhAtfA, forward primer.
14	5′‐CGGGATCCTTAGTGATGGTGATGATGATGTTTTCTAGTTCTGGCTCTCT	Cloning *MhAtfA* gene of *M. hydrocarbonoclasticus* DSM 8798 into pYLEX1 for construction of pYLEX1MhAtfA, reverse primer.
15	5′‐CTAGCTAGCGCTCTCCCTTATGCGACT	Amplifying *MaAtfA* expression cassette from pYLEX1MaAtfA into pYLP1A1GA to yield pYLP1A1GAMa, forward primer.
16	5′‐GCTCTAGAGAATTCGGACACGGGCAT	Amplifying *MaAtfA* expression cassette from pYLEX1MaAtfA into pYLP1A1GA to yield pYLP1A1GAMa, reverse primer.
17	5′‐CTAGCTAGCGCTCTCCCTTATGCGACT	Amplifying *MhAtfA* expression cassette from pYLEX1MhAtfA into pYLP1A1GA to yield pYLP1A1GAMh, forward primer.
18	5′‐GCTCTAGAGAATTCGGACACGGGCAT	Amplifying *MhAtfA* expression cassette from pYLEX1MhAtfA into pYLP1A1GA to yield pYLP1A1GAMh, reverse primer.
